# Bio-enriched composite materials derived from waste cooking oil for selective reduction of odour intensity

**DOI:** 10.1038/s41598-024-67302-4

**Published:** 2024-07-15

**Authors:** Anita Staroń, Jarosław Chwastowski, Magda Kijania-Kontak, Marek Wiśniewski, Paweł Staroń

**Affiliations:** 1 Department of Engineering and Chemical Technology, Cracow University of Technology, 24 Warszawska St., 31-155 Cracow, Poland; 2 Department of Civil Engineering, Cracow University of Technology, 24 Warszawska St., 31-155 Cracow, Poland; 3 Faculty of Chemistry, Nicolaus Copernicus University, 7 Gagarina St., 87-100 Toruń, Poland

**Keywords:** Waste cooking oil, Composites, Odour, Additive, Structure, Environmental sciences, Materials science

## Abstract

Currently, pathogenic microorganisms are becoming more active in public utility areas like parking lots and waste shelters due to the accumulation of organic waste. This uncontrolled waste leads to decay, altering its composition and presenting a microbiological risk to public health. Additionally, it emits unpleasant odors containing chemicals that irritate the mucous membranes, causing discomfort in the nose, throat, and eyes by stimulating the trigeminal nerve. These odors can have various negative effects on both quality of life and public health. The study investigated the physicochemical properties of oil composites enriched with natural additives and determined their effectiveness in reducing the intensity of nuisance odours. The research showed over 82% reduction in decaying meat odour and almost 65% reduction in ammonia odour. A higher impact of the given composites on reducing the odour from decaying meat than from ammonia was observed. This may be due to the biocidal properties of the additives used (turmeric, thymol, salicylic acid, hops and curly sorrel) and the higher intensity of ammonia odor compared to meat-derived odour. Despite the non-porous nature of the solids tested (with similar specific surface areas ranging from 0.66 to 0.88 m^2^/g), they were capable of sorbing NH_3_.

## Introduction

Currently, there is a strong development of disease-causing pathogens in public places, such as parking lots, passenger service areas, or local waste container shelters. The high microbiological risk is caused by the presence of organic waste in these places, such as food remains, which are left uncontrolled in places not intended for this purpose. The fractional analysis of municipal waste shows that food waste of plant origin may constitute up to 20%, animal waste up to 5%, and other organic waste up to 30%^1,2^. Some of them undergo putrefactive processes, change their consistency, cause sanitary and epidemiological threats and emit unpleasant odours. They consist of a mixture of volatile chemicals, both inorganic and organic, which are called odorous gases. These gases are detectable even at very low concentrations^3^. These substances include chemical compounds that, by stimulating the trigeminal nerve, can even irritate the mucous membranes of the nose, throat and eyes^4^. Unpleasant odours can have various effects on the quality of life and health, including headaches or breathing problems. Unpleasant odours are produced largely by industrial and agricultural activities, including animal breeding, rendering plants, sewage treatment plants, waste processing or disposal plants, paint shops, oil refineries, pulp and paper factories, as well as various sectors of the chemical industry. The emission of this type of pollutants into the atmosphere may result in, for example, deterioration of environmental quality and discomfort. Developments in the field of odour treatment are driven by advances in public awareness, national and international policies, and management and engineering approaches^5^.

Odour reduction can be done in three main ways, using chemical, physical or biological processes. Chemical odour reduction mainly involves ozonation and thermal or catalytic oxidation reactions. The most commonly used oxidants to remove unpleasant odours are ammonium, potassium and sodium perchlorate, sodium and potassium chlorate, sodium peroxide and perborate, hydrogen peroxide and their mixtures^6^.

Physical reduction of odour concentration is based on the processes of condensation, adsorption and absorption, usually using activated carbon, charcoal and permanganate-coated aluminum oxide. This method is of average efficiency due to the speed of saturation of the adsorbent^7,8^. The use of biological odour reduction methods is more ecological than the use of physicochemical technologies because they do not require the use of chemicals. An important advantage of biological methods is the ability to carry out processes at moderate temperature (10–40 °C) and atmospheric pressure. In the case of biological methods, bioreactors, biofilters, trickling biofilters and bioscrubbers are used^9^. The mentioned odour reduction methods mainly concern industrial odours. In open public spaces, removing unpleasant odours is more complicated. The solution could be anti-odour building materials, but unfortunately, the range of materials with odour-reducing properties in the construction industry is scarce, and when you narrow your search to solid anti-odour materials, you encounter a gap. Due to the growing interest in composite materials made from waste cooking oil, an attempt was made to modify their compositions. The desired effect of this procedure is the acquisition of new functional properties, such as a reduction in odour intensity.

A composition was developed in which materials based on waste cooking oil enriched with substances of natural origin with biocidal properties were produced, while emphasizing the complex structure of the pore-rich surface. The premise of this solution was to reduce the number of pathogenic microorganisms, what should result in reduced odor intensity or absorption of odour gases by the surface of the composites. The materials were designed in response to a gap in the building/construction materials market, where materials that absorb and neutralize odors from the environment are sought.

The work aimed to investigate the odour-reducing properties of oil composites enriched with turmeric, thymol, salicylic acid, hops and curly sorrel and to propose odour removal mechanisms.

## Materials and methods

### Process of obtaining oil composites

Oil composites enriched with natural additives were obtained from waste cooking oil (WCO), sand, acid catalyst and additives with antimicrobial properties (turmeric, thymol, salicylic acid, hops and curly sorrel). WCO came from a city restaurant, where it was used for thermal processing of food, so solid particles were removed from it by filtration before the process. Quartz sand with a grain size of 0.5–1.4 mm (air-dry condition) constituted the aggregate. Sulfuric acid (VI) (pure for analysis) was used as a catalyst. Hops and curly sorrel were commercial products in the form of dried; thymol and salicylic acid are white powders (pure), ground turmeric intended for food purposes. WCO was mixed with sulfuric acid using a planetary mixer for 5 min, then the aggregate was added (another 5 min of homogenization), and the additive. The mixture was mixed for another 5 min, transferred to aluminum moulds and shaken on a vibrating table to thicken. The mixtures were heated according to the experimental plan. After cooling, the composites were subjected to strength tests, based on which materials were selected for further tests. Table 1 shows the process parameters for obtaining oil composites containing natural additives. In the abbreviations used for samples, the number indicates the preparation of the composite according to Table 1, and the letter indicates the type of natural additive used: T—thymol, TU—turmeric, SA—salicylic acid, H—hops and CS curly sorrel.

**Table 1:** Process parameters for obtaining oil composites enriched with thymol, turmeric, curly sorrel, hops and salicylic acid.

Sample		H_2_SO_4_/WCO_cat_	Additive [%]	Temperature [℃]	Time [h]	WCO [%]
12	T, TU, SA, H, CS	0.24	1	190	20	25
13		0.04	7	210	20	20
15		0.04	1	210	20	25
16		0.24	7	210	20	25
22		0.14	4	200	19	25
33		0.24	7	210	18	20
35		0.24	1	210	18	25
37		0.24	7	190	20	20
41		0.24	1	210	20	20
42		0.04	1	210	20	25
43		0.24	7	210	20	25
44		0.14	4	200	18	22.5
51		0.24	4	200	19	22.5
61		0.14	4	190	18	22.5
68		0.24	1	190	19	22.5
74		0.14	7	200	18	22.5
80		0.14	4	210	18	22.5
85		0.14	7	210	18	22.5
86		0.14	7	210	19	22.5
88		0.24	7	210	19	22.5
92		0.24	1	210	19	22.5
93		0.24	4	190	17	22.5
96		0.24	4	210	16	22.5
97		0.24	1	210	16	22.5
98		0.24	7	210	16	22.5
96		0.24	4	210	12	22.5
97		0.24	1	210	12	22.5
98		0.24	7	210	12	22.5
99		0.24	4	210	12	22.5

### Testing the physicochemical properties of oil composites

Fourier-transform infrared spectroscopy was employed to examine the molecular structure of solid substances. The analysis was performed utilizing the Nicolet iS5 FT-IR spectrometer from Thermo Scientific, which covered the wavelength range of 500–4000 cm^−1^. To observe the surface structure of the oil composites, the scanning electron microscope Hitachi TM-3000 equipped with an energy-dispersive X-ray microanalyzer (EDS) was utilized. Mechanical strength tests were conducted using the Zwick-Roell Z600 testing machine, with an initial force of 25 N and a testing speed of 1 kN/min.

In order to determine the specific surface area and the affinity of the composites to ammonia, measurements of Low-Temperature Nitrogen and NH_3_ Adsorption Isotherms were carried out. The nitrogen adsorption isotherms were measured at 77.5 K using the Autosorp iQ gas adsorption apparatus (Quantachrome, USA). Before each measurement, the carbon samples were desorbed in a vacuum (below 10^–3^ Pa) at 323 K for 12 h. Measurement of NH_3_ Adsorption Isotherms was conducted at T = 298 K using a thermostat (with accuracy of ± 0.1 K) vacuum apparatus designed for gravimetric measurement of sorption isotherms. The NH_3_ equilibrium pressure was measured using the baratron transducers (MKS Instruments Germany, working in three ranges i.e. up to 1000 Pa, up to 10^4^ Pa and up to 1.33 10^5^ Pa). The samples were desorbed under vacuum at 323 K for 12 h under a pressure lower than 10^–2^ Pa. Next, the proper measurement was performed. According to Hook’s law, the change in the length of the quartz spiral, S (measured using a cathetometer) is equivalent to the change in the mass of the sample. The total change in the spiral length is the measure of NH_3_ adsorbed amount.

### Odour intensity testing

**Figure 1:**
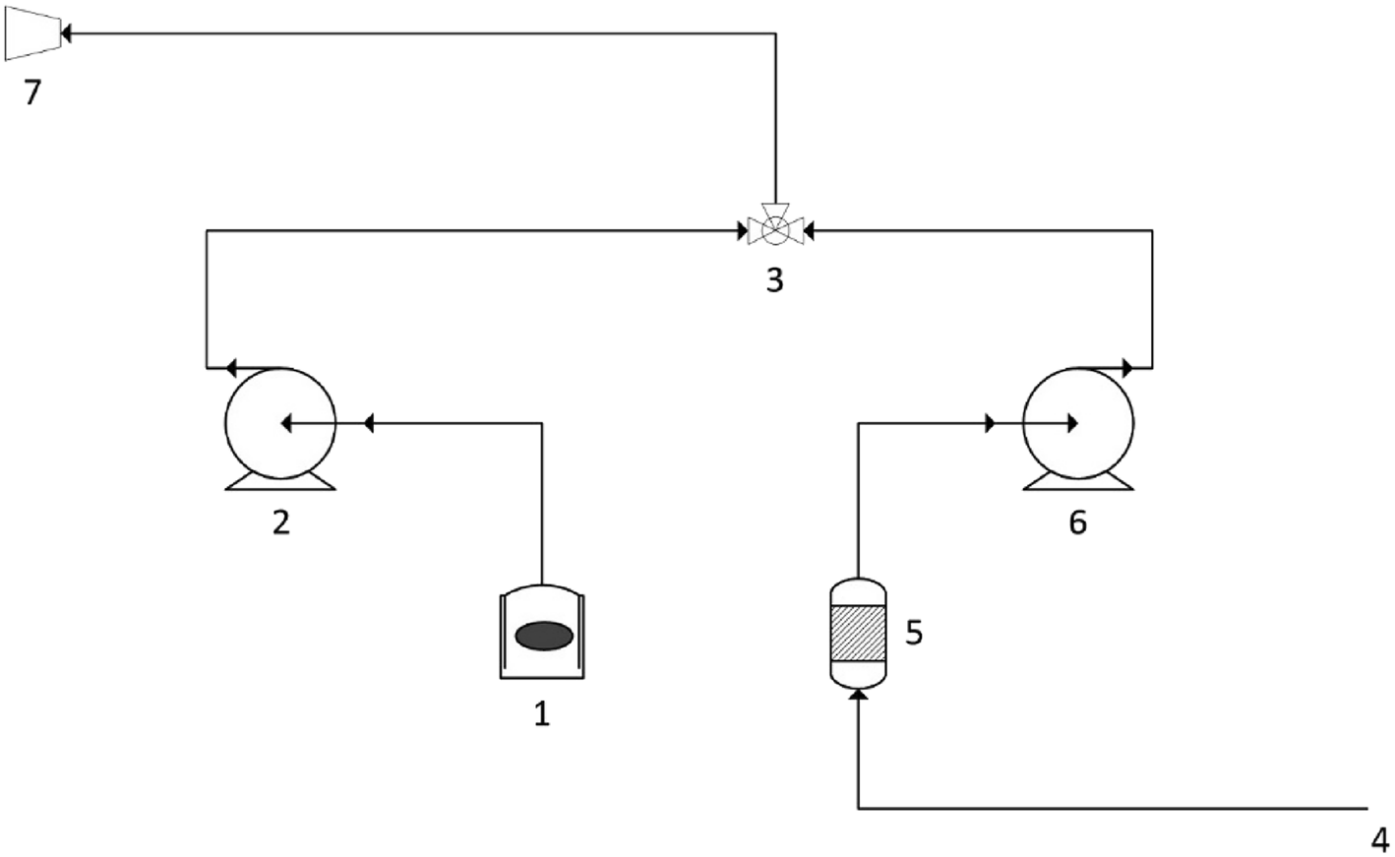
Schematic diagram of the station for determining the odour concentration using the dynamic dilution method: 1—container with the sample to be determined, 2—peristaltic pump for determining the sample odour concentration, 3—mixing valve, 4—source of odourless air, 5—carbon filter, 6—peristaltic pump for dispensing odourless air, 7—sniffing mask.

The odour intensity reduction was tested using the dynamic olfactometry method, in accordance with the PN-EN 13725:2022-07 standard. The odour sample in the container was connected to a peristaltic pump. During olfactory assessments, the tested sample was diluted with clean atmospheric air. Calibration of peristaltic pumps allowed us to determine the level of odour dilution from the imperceptible level to the moment when each study participant smelled the odour. Figure 1 shows a schematic diagram of the determination of odour concentration using the dynamic dilution method.

**Figure 2:**
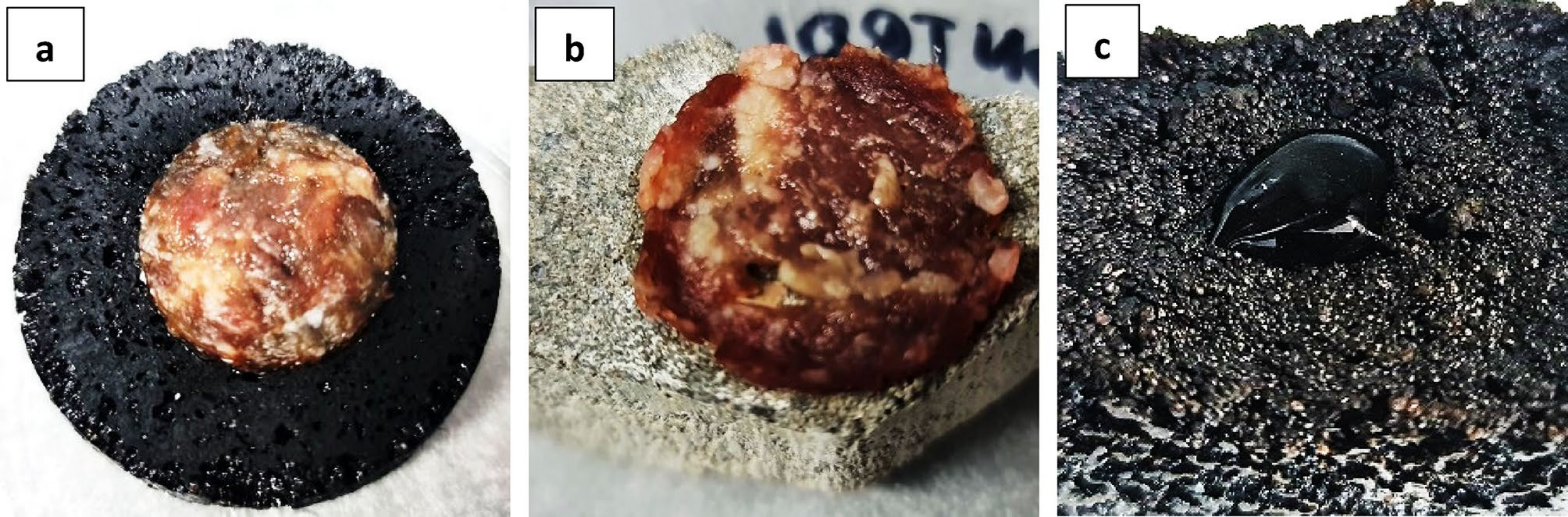
Samples for odour reduction tests: (**a**) oil and meat composite after 48 h; (**b**) paving stones (control) with meat after 48 h; (**c**) oil-ammonia composite.

During the odour intensity reduction test, the flow rate of clean, odourless air was constant and amounted to 750 cm^3^/min, and the variable parameter was the intensity of the odourant gas controlled in the range of 5–55 cm^3^/min. Cod odour concentrations were calculated as the geometric mean of the set of n all individual ZITE odour concentrations for a given material, and the odour reduction was related to the control sample. Based on the specific concentration and odour emission values, using selected mathematical tools, it is possible to estimate the scope of impact of odours emitted from a selected technological facility or selected materials that may cause odour nuisance. The tests were carried out in two variants differing in the source of odour emission. The first variant was connected to decomposing meat and the second to ammonia. Decomposing meat is rich in proteins, which hydrolyze and deaminate during decomposition, leading to the formation of amines, amino acids, hydrogen sulfide and other compounds. These compounds are responsible for the characteristic putrid odour. Ammonia is a byproduct of protein metabolism and in large quantities can be toxic. In addition, it is a compound with a very characteristic pungent odour that is easily recognizable even in low concentrations. In the first variant, the impact of oil composites enriched with natural additives on reducing the emission of unpleasant odours during the process of meat decomposition on their surface was assessed. 2 g of meat was placed on the paving stones (control) and the oil composite (Fig. 2). The meat (M) was placed in such a way that it had the same mass and contact surface with the tested materials. The prepared samples were incubated in containers at 22 °C for 48 h (Fig. 2a,b).

In the second variant, the source of the unpleasant odour was ammonia. 0.2 cm^3^ of 25% ammonia solution (A) was dosed onto the paving stones (control) and the tested material (oil composite) (Fig. 2c). The prepared samples were incubated in containers at 22 °C for 30 min. After this time, olfactometric tests were carried out to obtain individual odour concentrations based on the dilution values up to the detection threshold indicated by the evaluators.

The procedure for determining odour concentration (Cod [ou/m^3^]) from sources organized using the dynamic olfactometry method is described in detail in the PN-EN 13725:2007 standard.

The individual ZITE odour concentration [ou/m^3^] for a given material was calculated based on the dilution values up to the detection threshold D/T (V_clean_/V_tested_) indicated by the evaluators using the equation: $Z_{ITE} = \left( {Z_{NO} \cdot Z_{YES} } \right)^{0,5}$

where Z_NO_ = D/T + 1—indicates the D/T value when the odour is undetectable before the D/T value. Z_YES_ = D/T + 1—indicates the D/T value when the odour is detectable after the D/T value.

Odour concentration C_od_ [ou/m^3^], calculated as the geometric mean of the set of n all individual odour concentrations (ZITE) for a given measurement point according to the formula:

$$ C_{od} = \sqrt[n]{{Z_{ITE\_1} \cdot Z_{ITE\_2} \cdot Z_{ITE\_3} \cdot Z_{ITE\_4} \cdot \ldots \cdot Z_{ITE\_n} }} $$

where Z_ITE_n_—means the individual odour concentration according to the assessor.

### Statistical analysis of the results

The statistical analysis was performed using version 13 of STATISTICA by Tibco (TIBCO Software Inc., USA, www.tibco.com). In order to check the possibility of grouping the obtained research results based on the similarity of their characteristics, a cluster analysis was performed, which allows finding groups of data (clusters) in which objects in the same groups are more similar to each other than to those in other groups. Then, an analysis was performed using the k-means method, which divided the data groups so that the similarity in a given cluster was as high as possible and the separate clusters should differ from each other as much as possible.

## Results

### Physicochemical properties of composites enriched with natural additives

**Figure 3:**
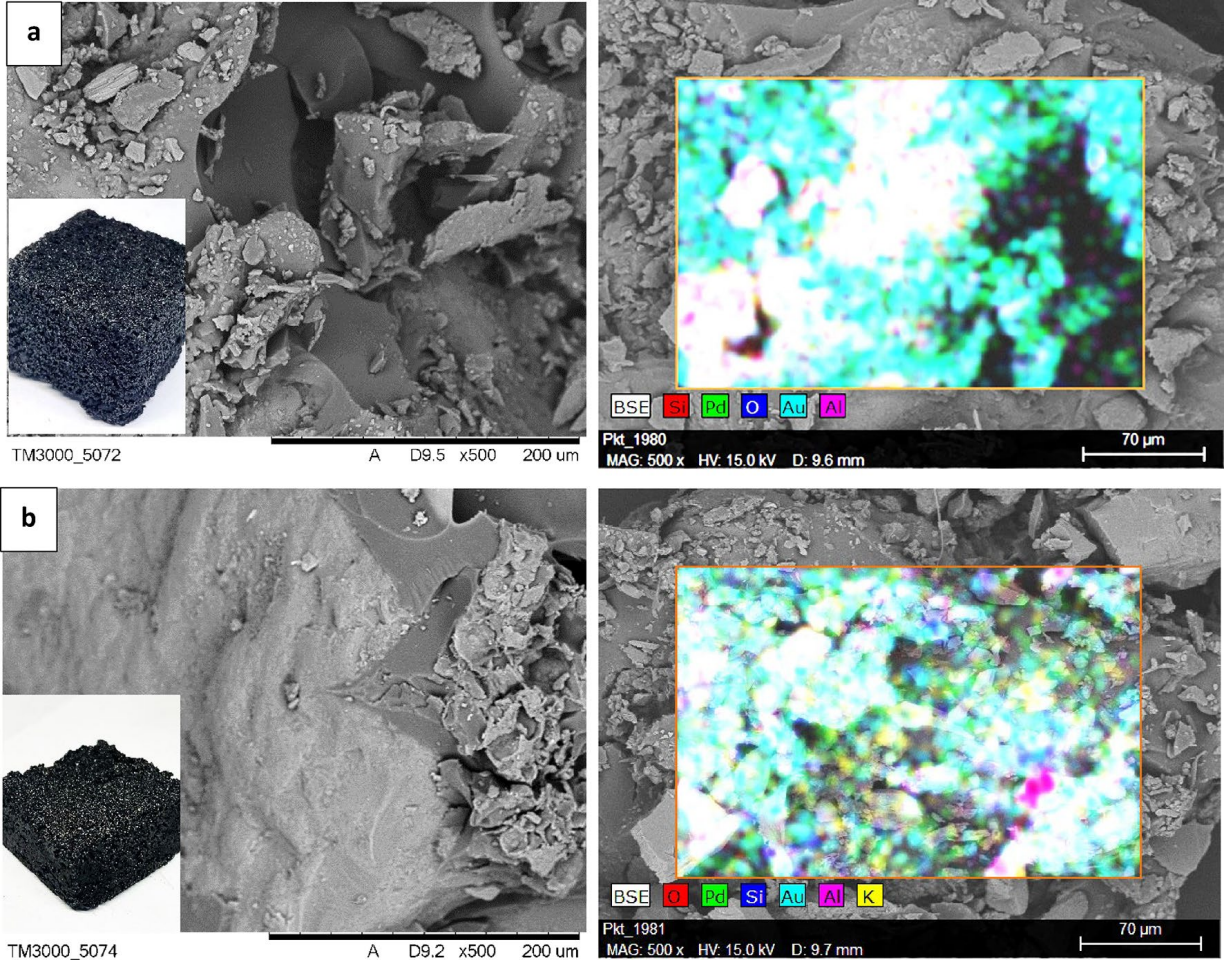
Photographs and SEM–EDS micrographs of the oil composite: (**a**) 16CS; (**b**) 35SA.

The surface of oil composites is heterogeneous, cracks, pores and sharp edges are visible. Sand components such as silicon, aluminum and calcium were identified on the surface of the oil composites, as was sulfur from the acid catalyst. Additionally, as a result of the sputtering of the samples, the presence of gold and palladium was found. Figure 3 shows photographs and SEM micrographs of composites No. 16CS and 35SA.

**Figure 4:**
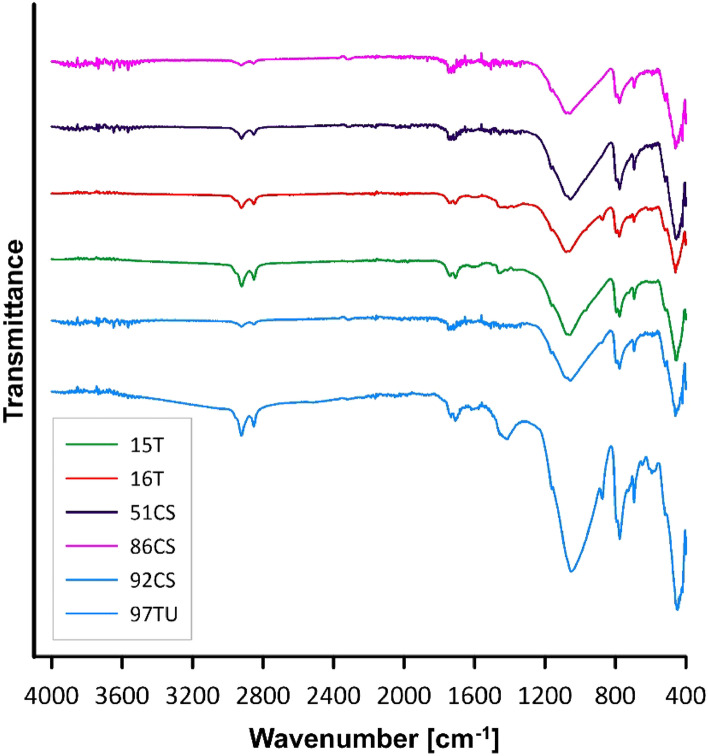
FT-IR spectra of selected oil composites.

The FT-IR spectra of oil composites resemble the spectrum of waste cooking oil^10^ however, differences are visible in the intensity of the peaks. Figure 4 shows the spectra of selected composites (16CS, 33SA, 16 T) and the characteristic peaks observed for all samples whose wave number is in the range of 2900–2850 cm^−1^, indicating the presence of both symmetric and asymmetric C–H stretching vibrations^11^. These vibrations suggest the presence of both aliphatic and aromatic compounds. Moreover, peaks in the wavelength range from 1700 to 1750 cm^−1^ were also observed for all spectra, which indicates the presence of stretching double bonds in the C=O group. The broad peak occurring in the wave number range of 1000–1100 cm^−1^ corresponds to the bending vibrations of C–O bonds^12^.

**Figure 5:**
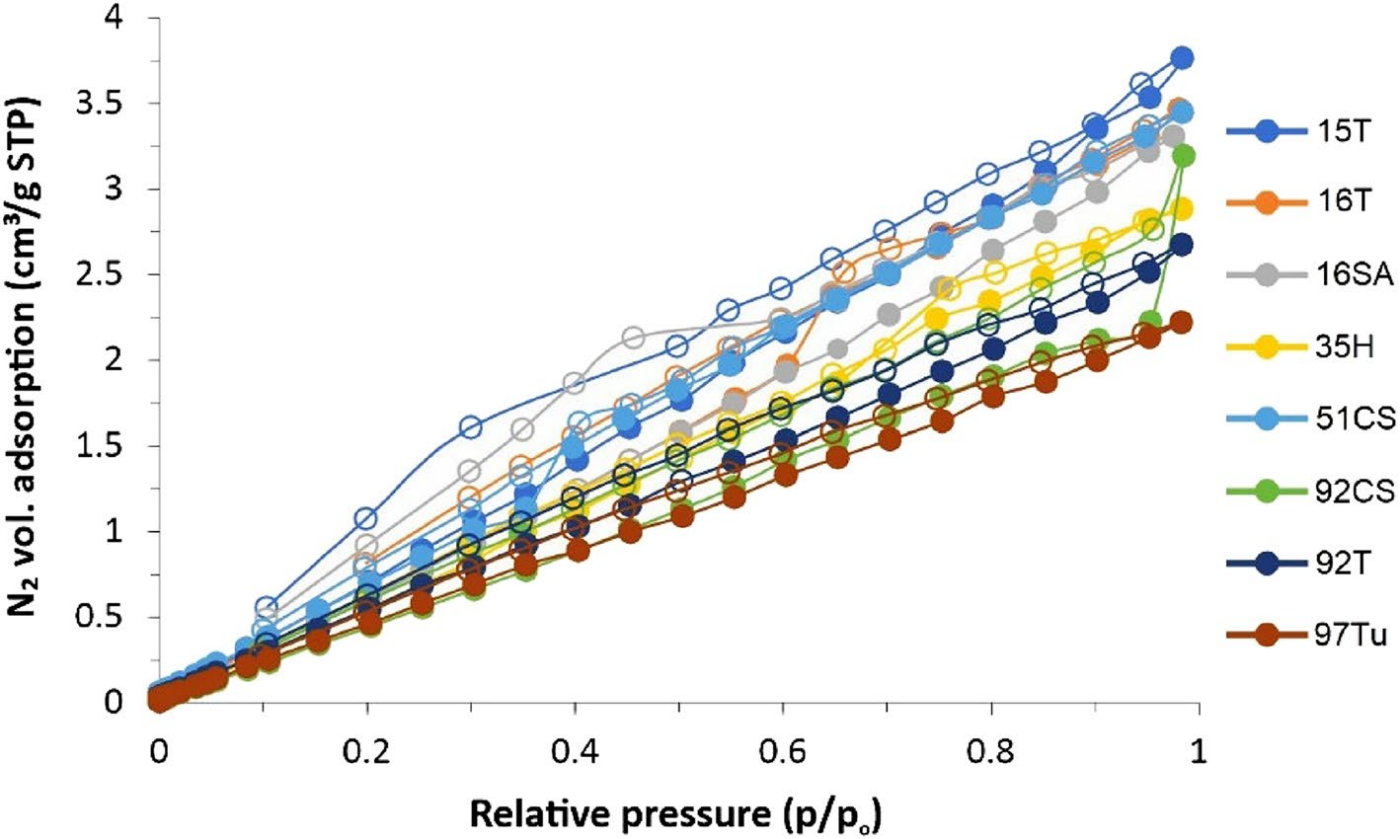
Low-temperature N_2_ adsorption isotherm. Full symbols, adsorption-, while empty- desorption branch.

The results from low temperature N_2_ adsorption measurements are collected in Fig. 5. The linear course of adsorption isotherms for all tested samples indicates the non-porous character of tested solids with similar specific surfaces in the range of 0.66–0.88 ± 0.2 m^2^/g.

Despite the lack of porosity, the tested materials can sorb NH_3_. The highest adsorption was measured for samples labeled 51CS and 97Tu while the lowest was for 16 T.

**Figure 6:**
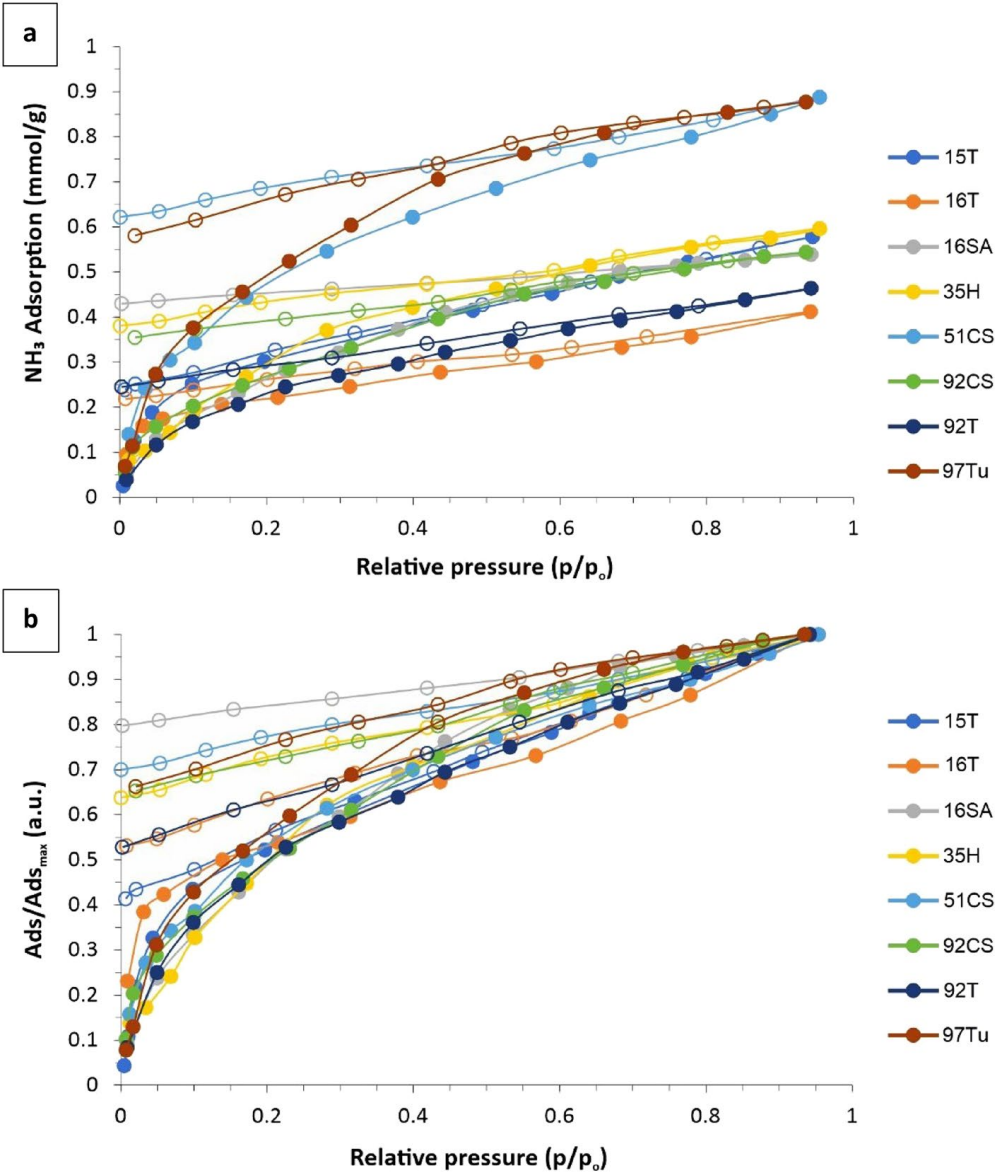
(**a**) NH_3_ adsorption isotherm measured at 298 K; (**b**) isotherms normalized to the maximal adsorption value.

The adsorption isotherms for all tested materials are of type I, which proves strong interactions of NH_3_ with the sample surface (Fig. 6a). The strong gas–solid interaction is also visible as a large hysteresis loop. The desorption branch (open symbols), starting from p/p_o_ ca. 0.6, differs significantly from the adsorption branch and is linear down to 0.1 p/p_o_.

The analysis of the A/A_max_ ratio (Fig. 6b) revealed that: (i) the sorption mechanism is similar for all tested samples—the course of the adsorption branches is very similar; (ii) the strongest NH_3_ interaction was observed for the 16SA sample—ca. 80% of NH_3_ remains on the sample surface; (iii) the weakest gas–solid interactions were observed for material labeled 15 T—ca. 40% remains adsorbed irreversibly.

**Figure 7:**
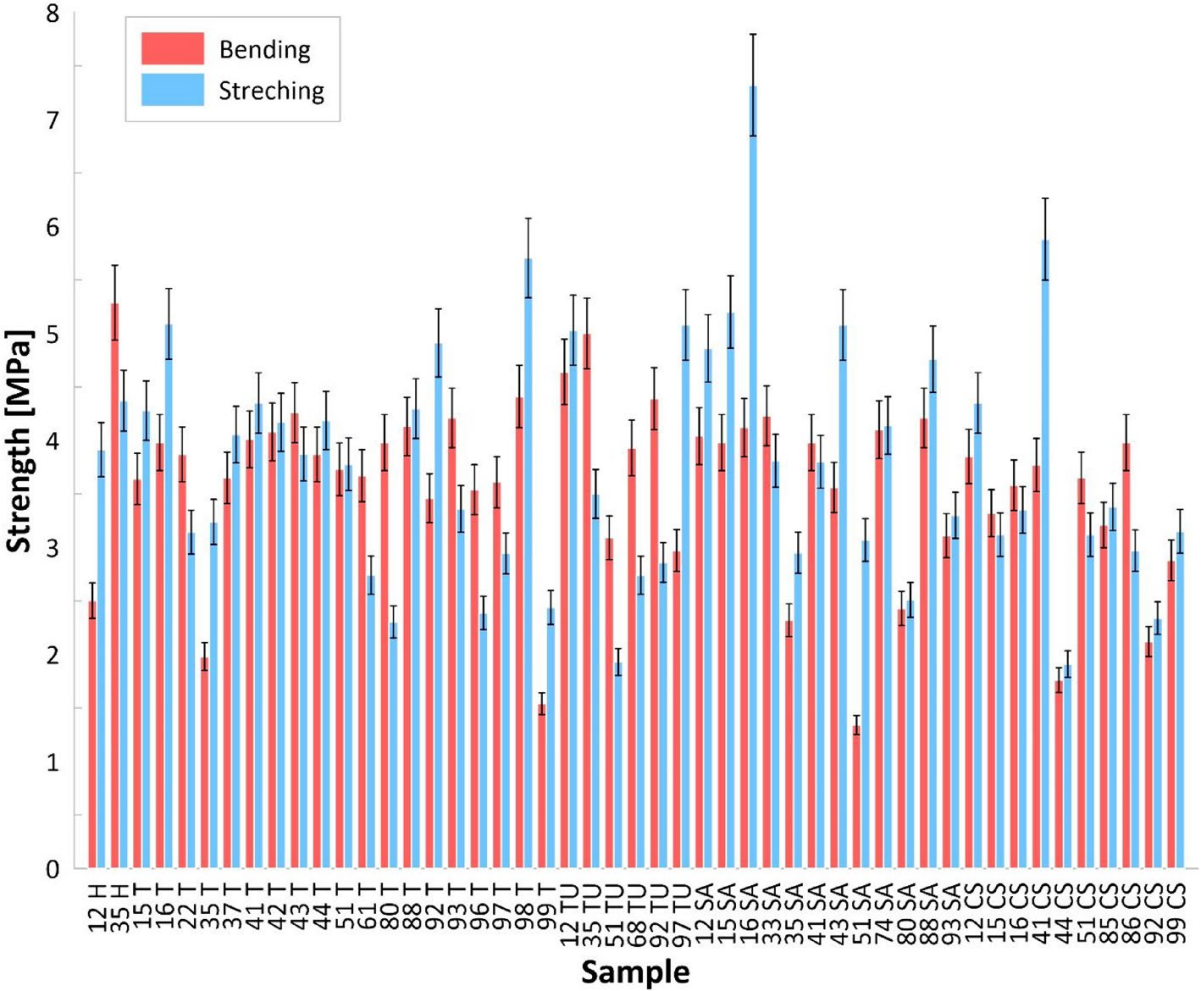
Results of bending and tensile strength tests of selected samples.

The results of the splitting bending and tensile strength tests are shown in Fig. 7.

### Reduction of odour intensity

**Figure 8:**
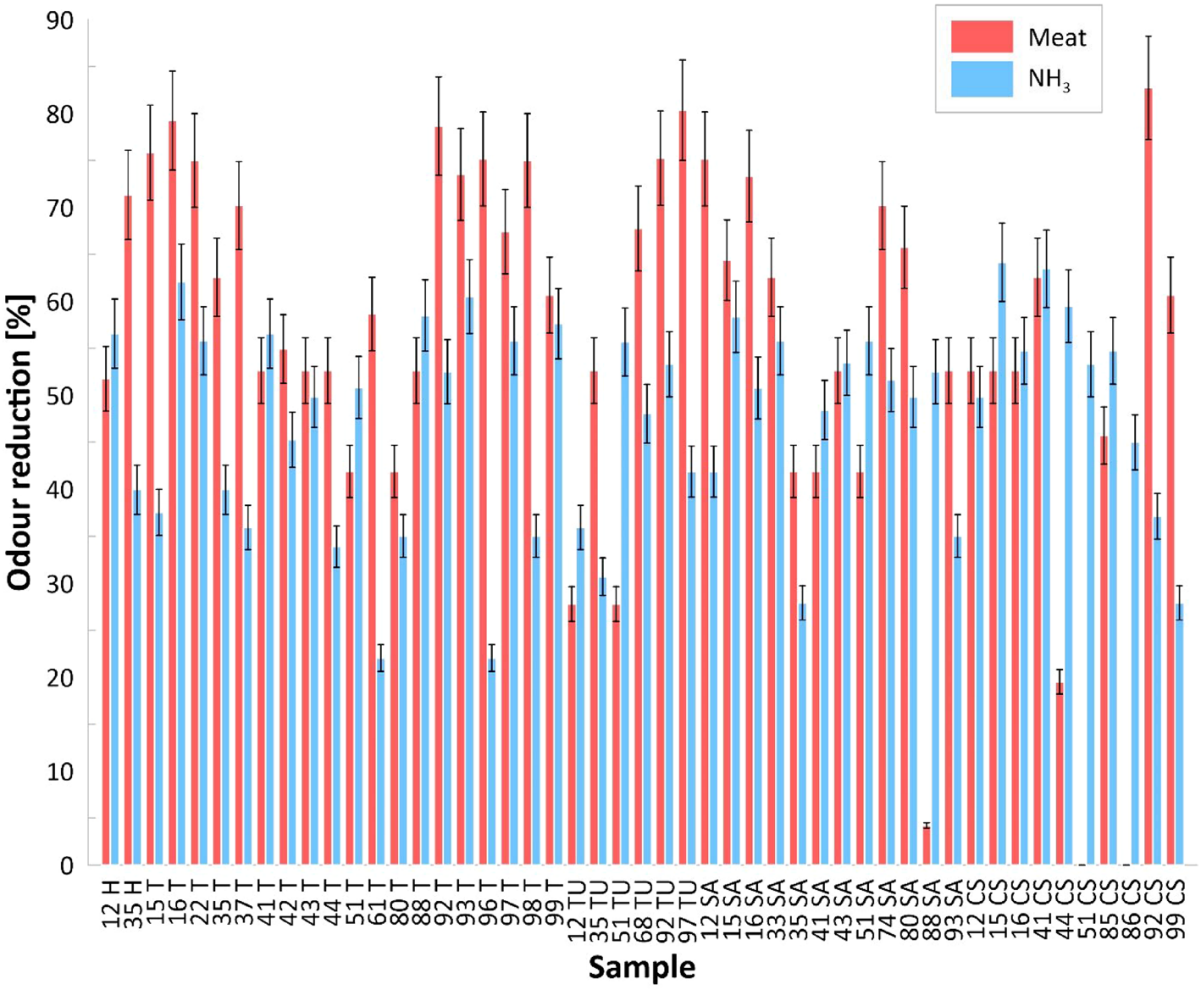
Graphical representation of the obtained odour reduction values.

In the case of tests conducted using meat as an odour source, a reduction in odour intensity of up to 82.73% was recorded, which was due to the diverse composition and structure of the composites. The highest reduction values were recorded for the 92CS material and the lowest for the 51CS and 86CS materials (no odour reduction). Studies conducted using ammonia as an odour source showed that the odour reduction ranged from 22.04 to 64.14% and was lower than in studies using meat. Figure 8 shows the odour reduction values for all composites for both experimental variants, with meat and with ammonia.

In the case of conducted research on the removal of ammonia odour in the form of vapours, it occurred through adsorption. The mechanism of ammonia adsorption to eliminate odour involves attracting ammonia molecules (NH_3_) to the surface of the adsorbent. Based on the fact that tested materials are non-porous the irreversible NH_3_ adsorption one can connect with presence of acid centers. The presence of surface carbonyl functionalities observed in FTIR spectra (Fig. 4) is not surprised in the WCO. Their Lewis acidity, perfectly corresponds with ammonia (NH_3_) which is a Lewis base^13^.

Moreover, the lack of porosity makes the tested systems highly selective materials for alkaline-amino-based odours.

In the first stage of odour removal, there is contact between ammonia molecules and the adsorbent acid centers. Van der Waals forces do not play a significant role in the interactions between ammonia particles and the surface of the adsorbent. The difference in electronegativity in carbonyl groups create stable dipoles, enabling the attraction of ammonia molecules. The material structure via torrefaction of organic matter^14^ provides numerous stable adsorption sites of various sizes, allowing for the effective trapping of ammonia molecules and reducing their content in the air, resulting in a decrease in odour levels. Furthermore, positively charged, hydrophobic surfaces promote increased efficiency in reducing odour nuisance. The presence of functional groups on the material's surface is a significant characteristic allowing for the binding of ammonia, as observed by analyzing the FTIR data for individual materials (Fig. 4)^15^.

There is no information in the literature on building materials that absorb odour. The parameter “odour intensity” is only a tool for assessing the relative degree of sustainability of building materials. On its basis, the negative odour of building materials is evaluated^16^. In order to give the materials odour-absorbing properties, their surfaces can be covered with coatings containing nanooxides, e.g. TiO_2_ or ZnO. Thanks to the photocatalytic properties of these oxides, the coatings have pollutant-degrading properties^17,18^. This type of solution requires a complicated procedure (producing a coating, applying it to the material and hardening it), and the introduction of nanoparticles into the environment is becoming increasingly controversial^19,20^.

### Influence of process parameters for obtaining oil composites on the effectiveness of odour reduction

**Figure 9:**
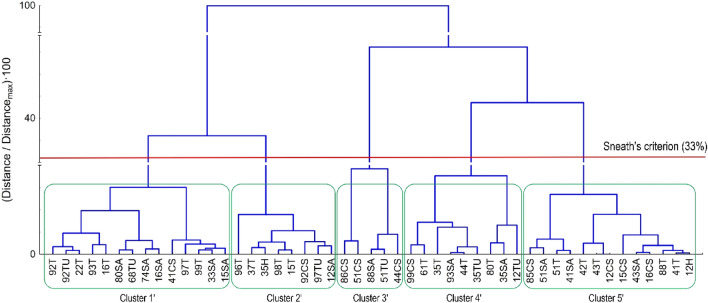
Dendrogram for odour reduction for materials (Ward's method, Euclidean distance).

The resulting data regarding odour reduction by the tested materials were subjected to cluster analysis (agglomeration and k-means). Cluster analysis grouped the composites according to the level of odor reduction. Dendrograms allow to visually determine the structure of data and identify natural cutoff points that indicate the most sensible number of clusters. Once the dendrogram is analyzed, it can be determined how many clusters are most appropriate for the data. This number of clusters can then be used as the k-value in the k-means method. The data were divided into five clusters using Ward's linkage method and Euclidean distance measurements. Applying the Sneath criterion with a more restrictive range (33%), five significant clusters were selected. The dendrogram constructed using cluster analysis, depicted in Fig. 9, illustrates the distribution of clusters.

**Figure 10:**
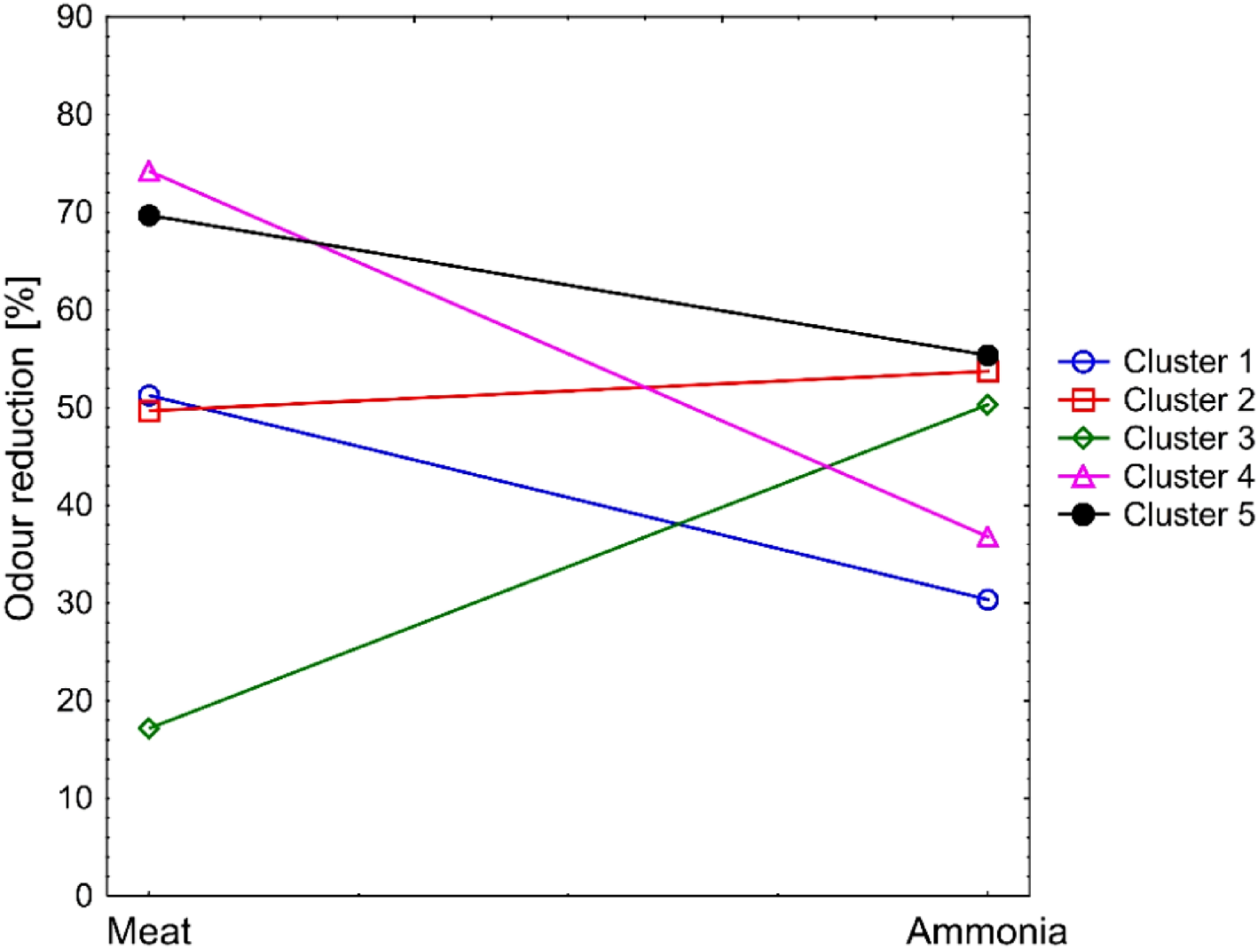
Graph of average odour concentration reduction parameters.

Based on the presented tree diagram, it can be seen that both the type of additive used and the parameters of the composite manufacturing process show similarities both to each other and to the type of odour reduced. The use of agglomerative analysis before applying the k-means method allowed for the identification of the optimal number of clusters using a dendrogram, and then enabled the accurate and effective assignment of objects to clusters using the k-means method. This combination of methods leads to more accurate and interpretable clustering results. According to the theory, the clusters obtained from the dendrogram analysis could show significant differences compared to the clusters determined using the k-means method and required the introduction of different markings. In the case of clusters derived from the dendrogram, the symbol (′) was used to distinguish them. Figure 10 shows a line chart of the k-means for individual clusters, and Table 2 shows the affiliation of cases to the appropriate cluster.

**Table 2:** Cluster elements.

	Cluster 1	Cluster 2	Cluster 3	Cluster 4	Cluster 5
Case	35K	12H	12K	12SA	15SA
	35SA	12CS	44CS	15T	16SA
	44T	15CS	51K	35H	16T
	61T	16CS	51CS	35T	22T
	80T	41SA	86CS	37T	33SA
	93SA	42T	88SA	92CS	41CS
	99CS	41T		96T	68K
		43SA		97K	74SA
		43T		98T	80SA
		51SA			92K
		51T			92T
		85CS			93T
		88T			97T
					99T

The data presented in the chart suggest dividing the clusters into two groups. The first group includes clusters 1, 4 and 5, while the second group consists of clusters 2 and 3. In the first group, it was observed that the tested materials had a lower ability to reduce the odour of ammonia compared to the reduction of the odour caused by meat decomposition. In clusters 2 and 3, samples containing dried curly sorrel root are observed, which may suggest that it absorbs ammonia. However, in the case of the second group of materials, an increase in the ammonia odour reduction capacity was observed. Group 5 was dominated by composites with thymol and salicylic acid, which showed strong biocidal activity and thus reduced the multiplication of bacteria on the meat surface. The average odour reduction values in relation to meat and ammonia are:

- *Cluster 1* Meat = 51.27%, Ammonia = 30.37%
- *Cluster 2* Meat = 49.71%, Ammonia = 53.73%
- *Cluster 3* Meat = 17.15%, Ammonia = 50.30%
- *Cluster 4* Meat = 71.96%, Ammonia = 35.47%
- *Cluster 5* Meat = 69.73%, Ammonia = 55.38%

The results of the analysis of variance (ANOVA), presented in Table 3, were used to assess the accuracy of the number of selected clusters. Based on the results obtained, it can be concluded that the clusters significantly (*p* < 0.05) reflect the nature of the variables.

**Table 3:** Analysis of variance.

No.	The between-group sum of squares	Degrees of freedom, df	Intra-group sum of squares	Degrees of freedom, df	Test-F	Test probability level, *p*
Meat	15362.75	4	2284.059	44	73.9868	0.000000
Ammonia	4503.82	4	1368.659	44	36.1975	0.000000

## Discussion

The reduction in odour intensity resulting from decaying meat is due to the biocidal properties of oil composites. Individual natural additives enriched in the composites have antimicrobial properties against selected bacterial strains^21–24^. Our previous studies have tested and confirmed the biocidal efficacy of oil composites containing thymol and salicylic acid against Gram-positive bacteria (*S. aureus* and *S. epidermidis*) and Gram-negative (*E. coli* and *P. aerugosa*)^25^. The highest antimicrobial activity against suspended *P. aeruginosa* bacteria (99% inactivation) was characterized by the composite containing 4% thymol. The composite containing 1% thymol caused a significant decrease in the viability of *E. coli* by up to 45%. In the case of *S. aureus*, thymol-containing composites also showed a strong biocidal effect of up to 70% (with 7% thymol). The addition of salicylic acid in the composites also resulted in bacterial reductions of 52% for *E. coli*, 99% for *P. aeruginosa*, 20% for *S. aureus* and about 25% for *S. epidermidis*.

Spoilage of meat products, e.g. sausages, is often the result of the development of aerobic spore-forming bacilli of the species *Bacillus subtilis, Staphylococcus aureus, Salmonella* Typhimurium or *Escherichia coli*^26,27^.When reviewing literature data on the impact of biological ingredients such as hops, thymol, turmeric, salicylic acid or curly sorrel on the antimicrobial properties of materials modified with them, one can notice their clear impact on reducing the proliferation capacity of selected bacterial strains responsible for meat spoilage. In research conducted by Bocquet et al.^28^, the high impact of hops as an antimicrobial factor was confirmed, significantly reducing the viability of 20 species of gram-positive bacteria. In the study by Kavoosi et al.^29^, scientists used thymol as an additive to gelatin films used as dressings. The conducted diffusion and plate tests showed a positive effect of the additive on reducing the viability of selected gram-positive bacteria (*B. subtilis, S. aureus*). Tests of turmeric as an addition to materials with antimicrobial properties were carried out, among others, by Antunes et al.^30^ showed that pure turmeric does not have antimicrobial properties against *Listeria monocytogenes* and *Salmonella* Typhimurium. The addition of ascorbic acid significantly increased the antimicrobial properties of pure turmeric. Salicylic acid has long been known as a factor limiting bacterial growth. Research conducted by Fang et al. on starch materials with the addition of salicylic acid against *E. coli, S. aureus* and *B. subtilis* bacteria suggests that the addition of 3% salicylic acid causes complete inhibition of the growth of selected microorganisms. Yildirim et al.^31^ in their research, they used extracts from curly sorrel (*Rumex crispus* L.) against *S. aureus, C. albicans, E. coli, B. sutbilis, P. aeruginosa*. The results clearly indicate the influence of these extracts on the growth capacity of selected microorganisms, in particular gram-positive bacteria. As in the previous cases, no significant impact of the tested extract on the reduction of the proliferative capacity of gram-negative bacteria was observed.

The selected additives have an excellent bacteriostatic effect due to their impact on cellular functions. The general mechanism of their action is related to damage to the cell membrane, which leads to protein leakage, loss of cellular energy due to a change in the pH gradient, and even cell death^32^.

## Conclusions

Olfactometric tests carried out on the ability of oil composites with additives to reduce odour have shown that they can reduce the intensity of the odour emitted during meat spoilage and the odour coming from the ammonia solution. The tested materials demonstrate both antimicrobial and adsorption abilities. All tested composites (except material 51CS and 86CS in the case of a method I using meat) showed effectiveness in reducing odour. The use of antimicrobial properties resulted in a reduction in odour intensity ranging from 19.5 to 82.7% while using ammonia as the odour source reduced it from 22.0 to 64.1%. Moreover, statistical analysis of the data showed the division of materials into groups in which differences in their ability to remove ammonia were observed. The study on the elimination of ammonia odor in the form of vapors used a selective adsorption process. The mechanism of odor elimination by ammonia adsorption involves the attraction of NH_3_ molecules to the surface of the adsorbent. Due to the non-porous nature of the materials studied, the irreversible adsorption of ammonia can be associated with the presence of acid centers. The presence of surface carbonyl groups as Lewis acids leads to reaction with ammonia, which is a Lewis base. Moreover, the lack of porosity makes the composites tested highly selective in removing alkali- and amine-based odours.

The results of odour intensity reduction studies shed new light on oil composites based on waste cooking oil. They confirm the possibility of modifying oil composites and giving them new, useful properties, and at the same time make it possible to manage the waste in the form of solid materials.

## Data Availability

The data are part of a research project and may be shared only with the author's permission (Anita Staroń, anita.staron@pk.edu.pl).
